# The sound of speed: How grunting affects opponents’ anticipation in tennis

**DOI:** 10.1371/journal.pone.0214819

**Published:** 2019-04-15

**Authors:** Florian Müller, Lars Jauernig, Rouwen Cañal-Bruland

**Affiliations:** Department for the Psychology of Human Movement and Sport, Institute of Sport Science, Friedrich Schiller University, Jena, Germany; University of Exeter, UNITED KINGDOM

## Abstract

Grunting in tennis is a widespread phenomenon and whether it influences opponents’ predictions of ball trajectory—and if so, why—is subject of ongoing debate. Two alternative hypotheses have been proposed to explain why grunting may impede opponents’ predictions, referred to as the *distraction account* (i.e., grunts capture attentional resources necessary for anticipation) and the *multisensory integration account* (i.e., auditory information from the grunt systematically influences ball trajectory prediction typically assumed to rely on visual information). To put these competing hypotheses to test, in the current study we presented tennis players with a series of temporally occluded video clips of tennis rallies featuring experimentally amplified, attenuated, or muted grunting sounds. Participants were asked to predict the ball landing position. Results indicated that higher grunt intensities yielded judgments of longer ball trajectories whereas radial prediction errors were not affected. These results are clearly at odds with the distraction account of grunting, predicting increased prediction errors after higher intensity grunts. In contrast, our findings provide strong support for the multisensory integration account by demonstrating that grunt intensity *systematically* influences judgments of ball trajectory.

## Introduction

The interplay of auditory information and sports performance has increasingly attracted researchers’ attention (for a recent review, see [1]). Indeed findings indicate that auditory information has ubiquitous effects on motor performance. For example, movement related sounds seem to be an integral part of the mental representation of these movements [2, 3]. Additionally, auditory information may be used to improve performance [4–6] (see [7], for a review). Finally, auditory information has been shown to influence anticipation as well [8, 9].

As concerns research on anticipation, a particular sound, namely grunting in tennis, has been a source of intense debate. On the one hand, some argue that grunting constitutes a physiological necessity in order to hit the ball with maximum force. This notion is supported by experimental studies demonstrating that grunting helps increase stroke velocity [10, 11] (see also [12], Exp. 1, for similar findings in martial arts) without causing additional oxygen cost [13]. On the other hand, tennis professionals such as Roger Federer [14] and Martina Navratilova [15] have argued that players’ grunting distracts and irritates their opponents giving them an unfair competitive advantage, as illustrated by the following quote by Roger Federer: “I’m OK with it to a certain level, but I don’t like it if it’s too loud or it’s used in key moments. That becomes unsportsmanlike.” [14].

Both theories emphasizing the multisensory nature of human perception [16] as well as empirical evidence [17] suggest that players’ grunting may indeed influence opponents’ behavior. However, various mechanisms have been proposed to explain grunting effects on opponents’ performances (see also [17], p. 3-4).

### Potential mechanisms driving the effect of grunting on opponents’ performance

First, early findings demonstrated that auditory perception is closely related to players’ performance in tennis [18, 19]. In these studies, players’ actual performance in tennis matches [18] and in anticipating a ball’s trajectory [19] suffered if auditory information was blocked completely by wearing earplugs. Based on the notion that “sound provides information about an interaction of materials at a location in an environment” [20], p. 8, Cañal-Bruland, Müller, Lach, & Spence [21] went beyond these all-or-nothing manipulations and demonstrated that the auditory information emanating from racket-ball contact (RBC) is actually used in predicting ball trajectories. RBC sounds that were experimentally amplified resulted in a) longer anticipated ball trajectories and b) strokes judged as more forceful than RBC sounds that were experimentally attenuated. This indicates that auditory information from RBC is integrated in the anticipation of ball trajectory.

It follows that grunting may, in fact, block (i.e., hide) the relevant auditory RBC information given that grunting temporally coincides with RBC, referred to as the *blocking account*. The very first study that empirically tested this *blocking account* was conducted by Sinnett & Kingstone [17]. Here masking the RBC sound with white noise (ostensibly mimicking an actual grunt) impacted players’ anticipation of the ball’s trajectory negatively. More recently, similar findings have been documented for judging the speed of volleyball smashes [22]. The authors showed that judgments’ accuracy suffered if auditory information was removed. Highlighting the importance of early auditory information in anticipation a recent study demonstrated that players anticipation of the landing position of volleyball smashes was determined largely by their acoustic properties [23]. These findings resonate with complaints from tennis professional Martina Navratilova that “the grunting prevented her from *hearing* [emphasis added] the ball” [24]. Note that even though Sinnett & Kingstone [17] opted to place the grunt such that it blocked the RBC sound, the authors acknowledge that the grunt can also be a temporally separate event (i.e., not overlap with RBC, see [17], p. 3).

In these cases, a second mechanism driving grunting effects on anticipation has been put forward—the *distraction account* [17]. The authors propose that the grunt itself may capture players’ attention at a time where it is required for both the visual perception of the ball’s trajectory as well as the processing of the RBC sound. After all, grunts are highly salient auditory stimuli having been measured at sound intensities in excess of 100 dB [25]. That grunting may indeed increase response time and accuracy in anticipating opponents’ actions has recently been demonstrated [12]. In this study, participants had to judge whether videos displaying moves of martial arts athletes depicted either high or low kicks. Videos edited to contain a simulated grunt resulted in longer reaction times and reduced accuracy (compared to original videos). In a similar vein, an experiment by Farhead & Punt [26] asked participants to judge the speed of actual tennis serves by tennis professional Maria Sharapova, who has been reported to produce grunts in excess of 109 dB [27]. Participants were more accurate in judging serves’ speed when Sharapova’s signature grunt was edited out.

Finally, grunting might influence players’ anticipation by virtue of a third process, namely *multisensory integration*. Established research has documented that perception of object properties is influenced by the joint contributions of different sensory modalities (for an overview see [28]). For example, tactile perceptions of roughness are influenced by the accompanying sounds produced by touching a surface [29, 30], and taste sensations of crispness are biased by the loudness and frequency composition of the accompanying sounds [31, 32]. Similarly, the systematic influence of the RBC sound on anticipated ball trajectory and perceived stroke intensity in tennis represents an example of such multisensory integration effects [21]. Based on findings indicating that grunting helps increase stroke velocity [10, 11] it seems reasonable to assume that grunting sounds convey information about the force of a given stroke. Following a central tenet of multisensory perception—that “multiple sources of information […] should be combined to yield the best estimate of the external property” [28], p. 731—one would predict that this very information should thus systematically influence players’ anticipation of ball’s trajectories. Specifically, strokes accompanied by grunting should be judged as more forceful, in turn resulting in estimates of longer ball trajectories.

We want to emphasize that these three mechanisms are by no means mutually exclusive, that is, more than one process might influence the anticipation of ball trajectories. However, inspection of actual tennis rallies featuring grunting players revealed that it is quite common that grunting does in fact *not coincide* with RBC. Therefore, in the rallies chosen for the current study, players’ grunts were audible only after the ball was hit, therefore ruling out the blocking account, at least for a substantial number of grunting occurrences. This leaves two alternative explanations for possible effects of grunting on the anticipation of balls’ trajectories—the *distraction account* and the *multisensory integration account*. Importantly, these two accounts yield different predictions. The *distraction account* predicts a general (i.e., unspecific) reduction in players’ anticipation performance. That is, louder grunts should yield increased errors in anticipation, without specific bias in any direction. In contrast, the *multisensory integration account* predicts systematic changes in the anticipation of the ball’s trajectory depending on the intensity of the grunt. To the extent that louder grunts are associated with harder, more forceful strokes [21], one would expect them to yield comparatively longer anticipated trajectories.

The aim of the current research was to examine whether higher grunt intensities yield either a) unspecific increases in prediction error or b) systematic changes in anticipated ball trajectories. To test these competing hypotheses we presented tennis players with a set of video clips depicting tennis rallies and asked them to predict where on the court the ball would land. Additionally, we manipulated the intensity of the grunts accompanying the respective strokes. According to the distraction account increasing grunt intensities should yield increased distraction resulting in an unspecific increase of participants’ prediction error. In contrast, the multisensory integration account would predict more intense grunts to be associated with more forceful strokes resulting in longer predicted ball trajectories.

## Materials and methods

### Sample

Sample size was based on previous studies of the impact of auditory information on ball position judgments [21] reporting effect sizes of at least *d* = .8 for pairwise comparisons of different sound intensities. Assuming that manipulations of grunting intensity may yield similar effects, a conservative estimate of an effect of at least *d* = .7 indicated a sample of *N* > 19 in order to reach a power of.8 at *α* = .05). We therefore recruited a sample of 31 all male tennis players (Age: *M* = 24.6 years; *SD* = 4.5, Range 18–36) with a mean playing experience of 14.5 years (*SD* = 4.8, Range 5–26) and an average playing frequency of twice per week (*M* = 1.88, *SD* = 1.28, Range 0–5). All played at regional club levels at the time of experimentation.

The study was approved by the Ethical Commission of the Faculty of Social and Behavioral Sciences of the Friedrich Schiller University (approval number FSV 18/22) and participants provided informed consent.

### Materials

Presentation of the experimental materials was implemented using standard web technologies (html, javascript) and shown via Mozilla Firefox 61.0.1 on a laptop computer (Acer Aspire V3-771G, 17.3” screen) using a pair of HyperX Cloud headphones.

#### Videos

A set of 42 video clips (Length: *M* = 3305, *SD* = 271 ms; 1280 × 720, 25 fps) were extracted from footage of the 2012 Barcelona Open finals between Rafael Nadal and David Ferrer [33]. These clips started with the player in the top half (Nadal) hitting a ball which was then returned by the player in the lower half (Ferrer). In all selected videos the following final stroke of the player visible in the top half of the tennis court (i.e., Nadal) was accompanied by grunting. In order to create the experimental materials video clips were then edited as follows. First, careful analysis of the material revealed that Nadal’s grunting was sustained well beyond the point in time when the ball hit the ground. Thus, in order to prevent participants from gaining access to full visual information of the ball’s landing position we occluded the ball’s trajectory (i.e., any visual information) at 240 ms after the end of the RBC sound by replacing the clip’s video track with a solid white screen (mean duration = 340 ms; a total of three participants mentioned noticing a slightly longer grunt duration on the final stroke). The audio track continued until the end of Nadal’s grunt (mean length of complete clip = 3305 ms). Second, using video editing software Adobe Premiere Pro 3.0 the section of the audio track of each clip containing the grunt was either amplified by 8 dB (high), left at original intensity (normal), attenuated by 8 dB (low), or muted completely (off). This constituted the within subject factor *Grunt Intensity*, yielding a final set of 168 (42 videos × 4 sound manipulations) video clips that were shown to participants in individually randomized order.

#### Exit questionnaire

Participants answered a set of questions covering demographic information (e.g., sex, age), tennis related information (e.g., playing experience, current and maximum playing level), and questions concerning the design and hypotheses of the study.

### Procedure

**Fig 1:**
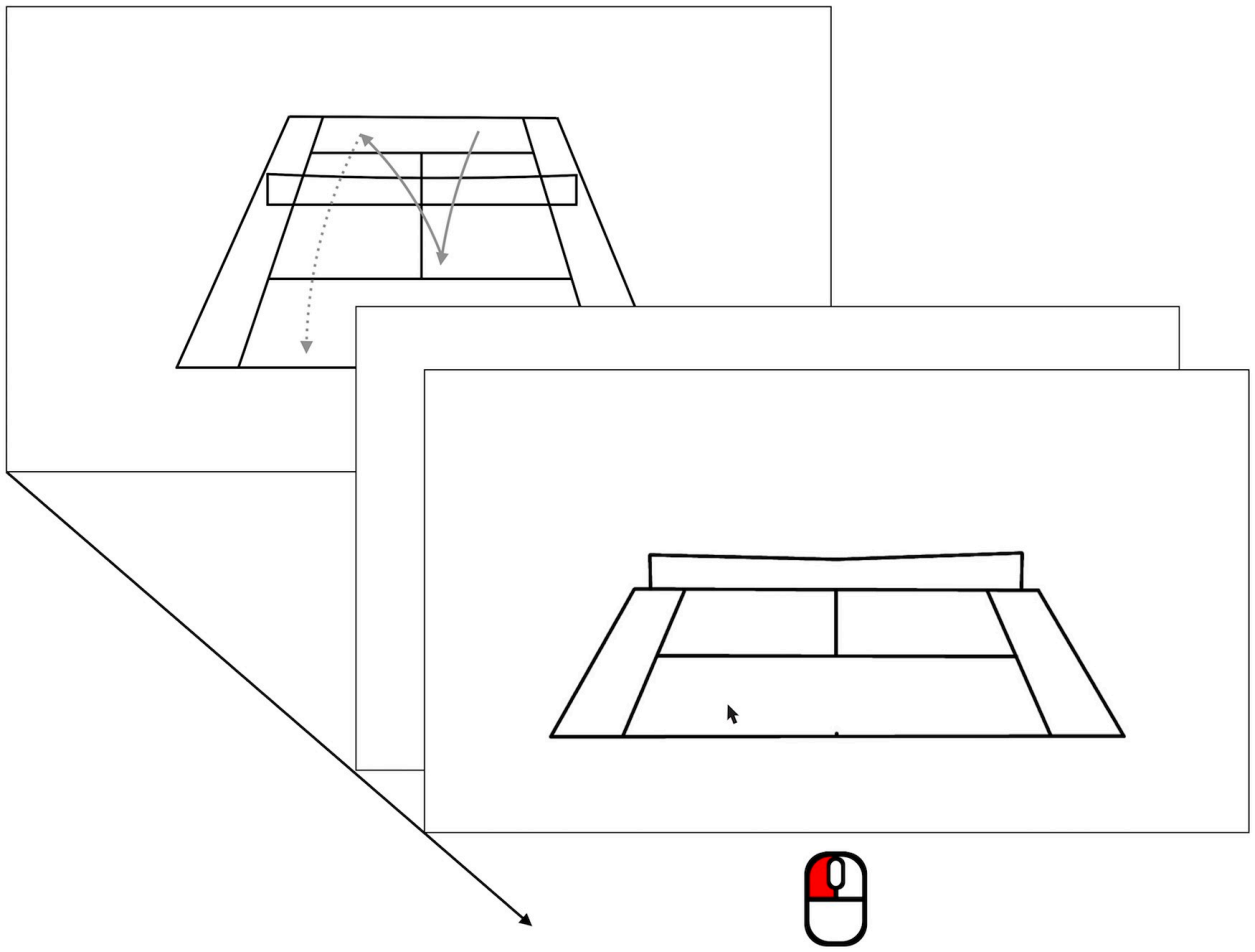
Presentation of the video clips and response collection. After each video clip showing a rally participants indicated the ball’s anticipated landing position via mouseclick.

Participants were tested individually in a small room on the premises of their local tennis club. Here they were seated in front of a laptop computer and were given a pair of headphones to wear during the experiment. All further instructions were given on screen. Specifically, participants learned that they were to view a number of short video clips of tennis rallies. After each clip a schematic outline of the just seen tennis court was shown, and participants were to indicate the estimated landing position of the ball by clicking in the appropriate location (for an illustration, see Fig 1). Upon registering their judgment, the experiment continued by showing the next video clip. After completing 80 trials participants were offered the opportunity to take a short break. After all clips had been shown, the experiment concluded with an exit questionnaire assessing age and sex as well as data on their playing experience (years of playing, frequency of playing per week) and hypotheses or suspicions concerning the goal of the study.

## Results

**Fig 2:**
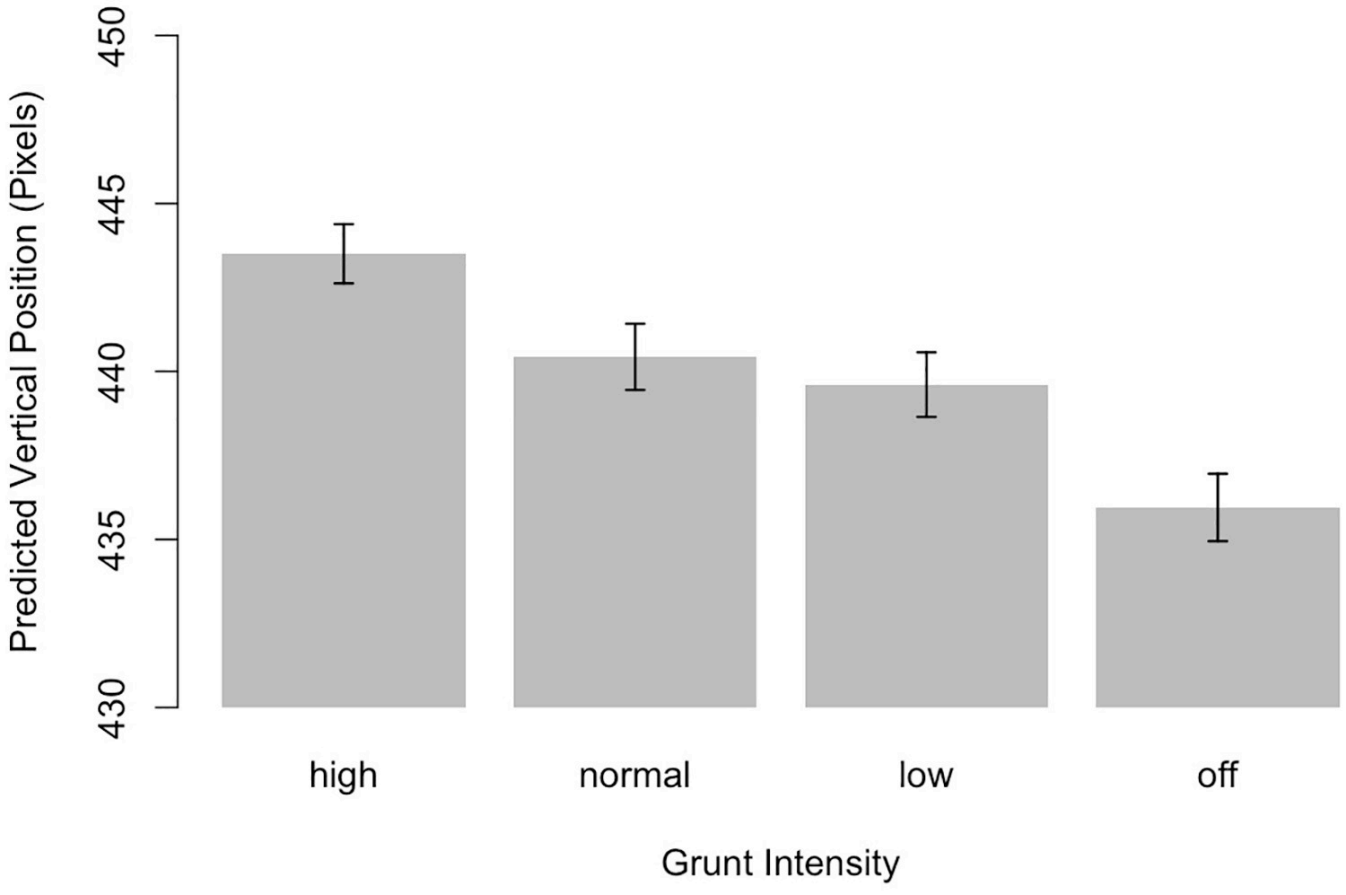
The influence of grunt intensity on judgments of the ball’s landing position in the longitudinal (i.e., vertical) axis. Error bars indicate 95% confidence intervals.

In order to test whether the manipulation of the grunts resulted in systematic shifts in the estimated lengths of the ball trajectories as predicted by the *multisensory integration account*, we subjected the vertical coordinates of participants’ estimated ball landing positions to an ANOVA with the within-subjects factor *Grunt Intensity* (high, normal, low, off). Results revealed a significant effect of *Grunt Intensity*, *F*(3, 90) = 30.24, *p* <.001, *η*^2^ = .5. Fig 2 illustrates that the louder the grunt, the longer participants predicted the ball’s trajectory to be.

Bonferroni corrected post-hoc comparisons (see Table 1) indicated that strokes with high intensity grunts resulted in significantly longer trajectories than normal grunts, whereas strokes with normal grunts did not differ from those with low grunts. Finally, low grunts yielded significantly longer trajectories than muted grunts. The predicted horizontal position was not affected by the grunt’s intensity, *F*(3, 90) = 1.0, *p* = .4.

**Table 1:** Pairwise comparisons for each level of grunt intensity.

	high	normal	low
normal	4.43*		
low	4.96*	0.97	
off	9.23*	5.14*	4.82*

*: *p* <.001 (t-values for dependent samples t-test)

**Fig 3:**
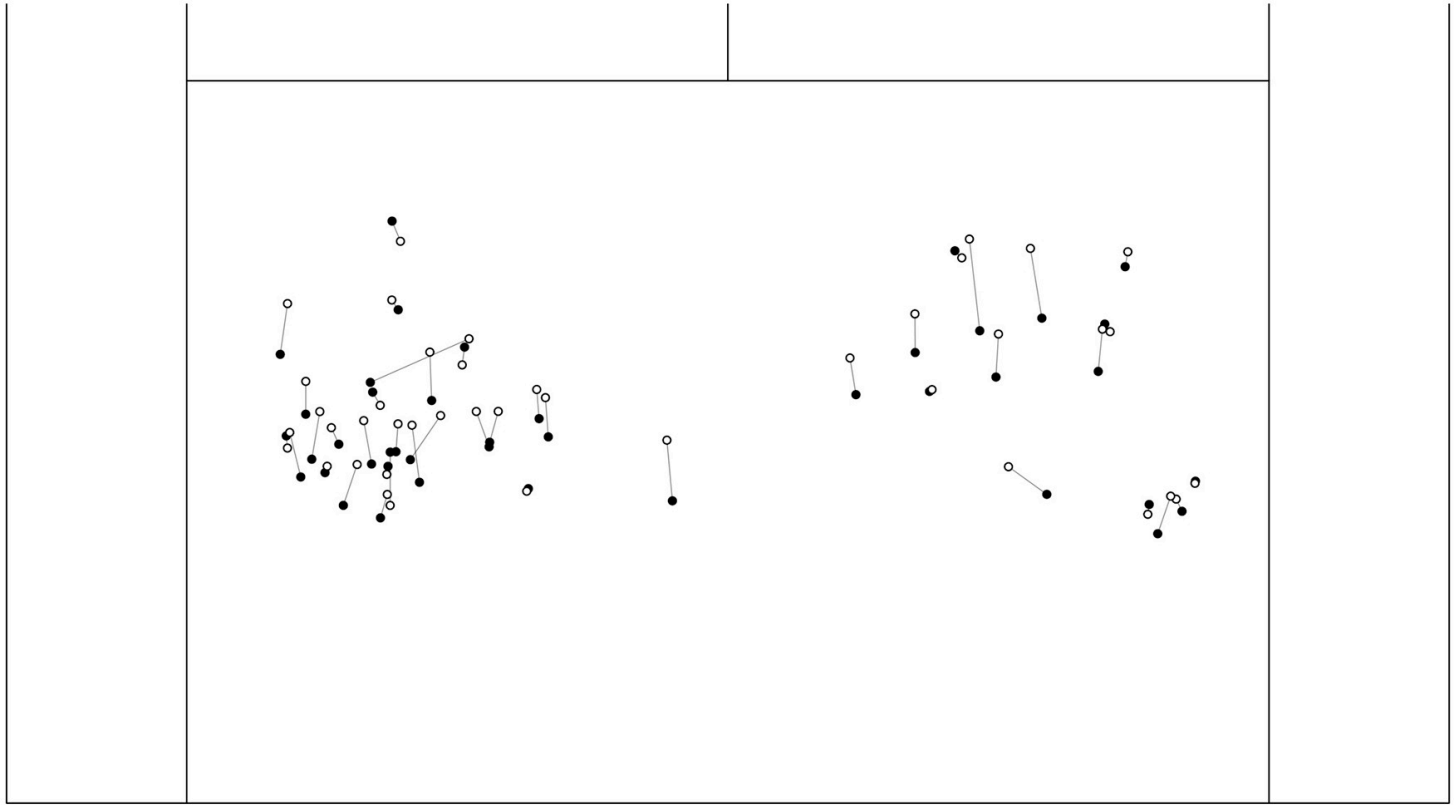
The influence of grunt intensity on judgments of the ball’s landing position on the court. Solid black dots = high intensity grunt; white dots = low intensity grunt.

In order to arrive at an estimate of these effects in terms of actual distance on the court we then subjected all pixel based coordinates to a homography transformation, mapping the coordinates of the schematic court outlines to the real world coordinates of an actual tennis court (per video homography matrices and subsequent coordinate transformations were computed using the OpenCV 3.4.4 library for Python). For purposes of clarity, Fig 3 illustrates the effect of grunt intensity on participants’ anticipated ball landing positions by contrasting predictions following high intensity grunts with those following low intensity grunts. On average high intensity grunts yielded predictions 18 cm closer to the baseline (i.e., the bottom of the screen) than low intensity grunts.

**Fig 4:**
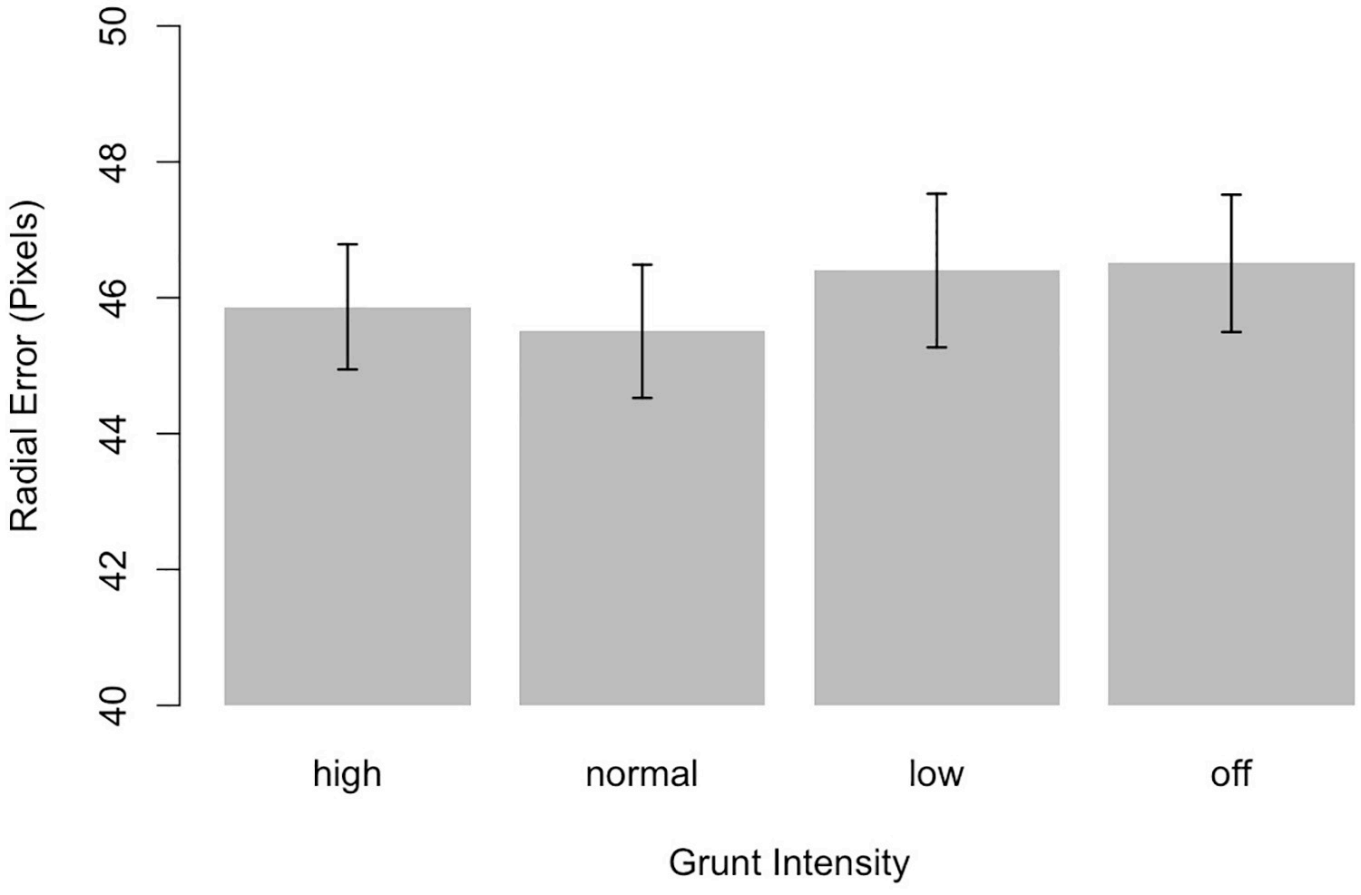
The influence of grunt intensity on radial error of the ball’s landing position. Error bars indicate 95% confidence intervals.

In order to test the predictions of the distraction account, i.e., the notion that louder grunts reduce participants’ accuracy in anticipating the ball trajectory we submitted participants’ radial errors to the very same ANOVA. Results revealed that radial errors were not affected by grunting, *F*(3, 90) = .62, *p* = .61, as illustrated in Fig 4. These findings are in contrast to the distraction account positing that grunts of higher intensity should increase players’ prediction errors.

Finally, in an additional exit questionnaire probing participants for possible hypotheses, a total of *N* = 8 participants mentioned either noticing differences in grunting intensity or a possible relationship between grunt intensity and stroke power or ball trajectory. We therefore repeated the previous analyses without those participants. However, this did not affect the statistical significance of any the previously reported results.

## Discussion

The aim of the study was to test whether tennis players’ grunting influences an opponent’s predictions of the ball’s trajectory. Specifically, we tested whether possible effects of grunting on anticipation were either in line with a distraction account (i.e., grunting distracts players during anticipation) or a multisensory integration account (i.e., grunting is integrated in and systematically affects the perception of a given shot). In line with a multisensory integration account results indicated that grunting led to systematic changes in predicting the ball’s trajectory, with louder grunts resulting in longer predicted trajectories. In contrast, predictions of the distraction account were not supported by the data: participants’ accuracy in anticipating the different shots was neither affected by the presence nor the intensity of grunting.

### Relationship to established findings

Going beyond established work on the role of visual perception in anticipation [34, 35] the current findings add to reports on the relevance of auditory information for tennis players’ performance in general [18, 19] and on the influence of auditory information emanating from the RBC in particular [17, 21]. However, previous studies on the effects of grunting have typically investigated situations where grunting temporally coincided with the sound of RBC [17, 26] (for related findings in volleyball, see [22]) making it difficult to tease apart effects of multisensory integration, distraction, and blocking. Of course, we do not suggest that the blocking account put forward by these studies (i.e., the notion that grunts block relevant auditory information) is irrelevant for the effects of grunting on anticipation. Rather, it may play a decisive role in mediating the effects of grunting in those situations where grunting actually coincides with the RBC. However, for those situations where grunting occurs after the RBC, the current findings are the first that (a) provide evidence on the influence of grunting on anticipation and (b) allow to differentiate between the mechanisms underlying grunting effects on anticipation.

### Practical implications

Our findings are also highly relevant for the ongoing debate on the role of grunting in tennis tournaments. To the best of our knowledge, the current work provides the first evidence for a systematic influence of grunting on shot anticipation. Even though grunting may not prevent players from “hearing the ball” (as argued by Martina Navratilova; see [24]) under these conditions, it nevertheless affects anticipation. However, rather than reducing accuracy in the judgment of balls’ landing positions in general, participants in the current study (all active club level tennis players) seemed to be able to integrate the auditory information from the grunt into their anticipatory judgments. Perhaps this is driven by the fact that they have become attuned to the relationship between grunting and actual transmitted force that has been documented in previous research [10, 11].

An interesting implication of our findings could be that players may deliberately change their grunting styles to deceive their opponents. Specifically, players could try to grunt profoundly when performing a soft lob or suppress grunting when performing a powerful smash therefore biasing opponents’ anticipation. However, whether the reported findings as well as the idea to deceive an opponent by using inconsistent grunts may generalize to actual performance on the court remains to be tested in future studies.
